# Comprehensive bulk and single-cell transcriptome profiling give useful insights into the characteristics of osteoarthritis associated synovial macrophages

**DOI:** 10.3389/fimmu.2022.1078414

**Published:** 2023-01-05

**Authors:** Shengyou Liao, Ming Yang, Dandan Li, Ye Wu, Hong Sun, Jingxiao Lu, Xinying Liu, Tingting Deng, Yujie Wang, Ni Xie, Donge Tang, Guohui Nie, Xiaoqin Fan

**Affiliations:** ^1^ Shenzhen Key Laboratory of Nanozymes and Translational Cancer Research, Department of Otolaryngology, Shenzhen Institute of Translational Medicine, The First Affiliated Hospital of Shenzhen University, Shenzhen Second People’s Hospital, Shenzhen, Guangdong, China; ^2^ Department of Otolaryngology, Shenzhen First People’s Hospital, The Affiliated Hospital of Jinan University, Shenzhen, Guangdong, China; ^3^ Guangdong Provincial Engineering Research Center of Autoimmune Disease Precision Medicine, the Second Clinical Medical College of Jinan University, the First Affiliated Hospital of Southern University of Science and Technology, Shenzhen People’s Hospital, Shenzhen, China; ^4^ Department of Otolaryngology, School of Basic Medical Sciences, Guangzhou Medical University, Guangzhou, Guangdong, China; ^5^ The Bio-bank of Shenzhen Second People’s Hospital, The First Affiliated Hospital of Shenzhen University, Shenzhen, Guangdong, China; ^6^ State Key Laboratory of Chemical Oncogenomics, Guangdong Provincial Key Laboratory of Chemical Genomics, Peking University Shenzhen Graduate School, Shenzhen, Guangdong, China

**Keywords:** osteoarthritis, synovium, immune infiltration, macrophage, Scissor analysis

## Abstract

**Background:**

Osteoarthritis (OA) is a common chronic joint disease, but the association between molecular and cellular events and the pathogenic process of OA remains unclear.

**Objective:**

The study aimed to identify key molecular and cellular events in the processes of immune infiltration of the synovium in OA and to provide potential diagnostic and therapeutic targets.

**Methods:**

To identify the common differential expression genes and function analysis in OA, we compared the expression between normal and OA samples and analyzed the protein–protein interaction (PPI). Additionally, immune infiltration analysis was used to explore the differences in common immune cell types, and Gene Set Variation Analysis (GSVA) analysis was applied to analyze the status of pathways between OA and normal groups. Furthermore, the optimal diagnostic biomarkers for OA were identified by least absolute shrinkage and selection operator (LASSO) models. Finally, the key role of biomarkers in OA synovitis microenvironment was discussed through single cell and Scissor analysis.

**Results:**

A total of 172 DEGs (differentially expressed genes) associated with osteoarticular synovitis were identified, and these genes mainly enriched eight functional categories. In addition, immune infiltration analysis found that four immune cell types, including Macrophage, B cell memory, B cell, and Mast cell were significantly correlated with OA, and LASSO analysis showed that Macrophage were the best diagnostic biomarkers of immune infiltration in OA. Furthermore, using scRNA-seq dataset, we also analyzed the cell communication patterns of Macrophage in the OA synovial inflammatory microenvironment and found that CCL, MIF, and TNF signaling pathways were the mainly cellular communication pathways. Finally, Scissor analysis identified a population of M2-like Macrophages with high expression of CD163 and LYVE1, which has strong anti-inflammatory ability and showed that the TNF gene may play an important role in the synovial microenvironment of OA.

**Conclusion:**

Overall, Macrophage is the best diagnostic marker of immune infiltration in osteoarticular synovitis, and it can communicate with other cells mainly through CCL, TNF, and MIF signaling pathways in microenvironment. In addition, TNF gene may play an important role in the development of synovitis.

## 1 Introduction

Osteoarthritis (OA) is the most common chronic joint disease in the population, which is mainly characterized by cartilage degeneration, subchondral bone sclerosis, osteophyte formation, and synovial joint inflammation. It can significantly alter joint dysfunction in older people and lead to disability and reduced the quality of life (1, 2). Furthermore, approximately 9.6% of men and 18% of women over 60 years old experience OA; 25% of patients with OA are regarded to have disabilities according to the World Health Organization (3). In addition, OA creates a tremendous socioeconomic burden worldwide (4). Although there have been multiple studies on OA formation and progression, the pathogenic mechanism and etiology of OA remains unclear. Therefore, it is necessary to understand the molecular mechanisms of OA and find effective therapeutic strategies to combat it.

Recently, more research has shown that the synovium plays an important role in the progression of OA (5, 6). Moreover, synovitis is associated with the pathological changes of OA, causing bone and cartilage destruction (4, 7). Molecular biology research has revealed that genes, such as long noncoding RNAs (lncRNAs) and mRNA dysregulation in the synovial tissue, are often associated with the pathogenic process of OA (8). Leukocyte infiltration, Th1/Th2-type cytokines, cellulose deposition, M2 Macrophages, and immune infiltration in the synovium were reported to play a critical role in synovitis and cartilage destruction in patients with OA (9–17). In particular, synovitis and inflammation have become research hot spots (18). The transcriptome data and integrated bioinformatics methods are widely used to study human diseases, revealing the cellular and molecular events of the diseases (19, 20). Previous integrated bioinformatical studies have revealed several hub genes, including SCRG1, ZNF160, and CCL5, that participate in the inflammation of OA, which may act as therapeutic targets for OA therapy. In addition, the cellular interaction relationships including NK cells, Macrophages, T cells, dendritic cell (DC), and key biological signaling pathways, including inflammation, immune response, osteoclast differentiation, bone development, and so on, have been clearly identified and validated (21–23). Notedly, fibroblast-like synoviocytes played an inflammatory role through TNF signaling pathway, and this pathway was considered as the key pathway involved in OA inflammatory development by targeting SELE, SERPINE1, and NFKBIA (24, 25). Overall, these findings provide a novel insight into the inflammatory factors or inflammatory signal molecules of OA. However, only few bioinformatics studies have solely focused on OA and its correlation with the molecular and cellular events of inflammation in synovial microenvironment.

To describe the cellular and molecular events and reveal its inflammatory pathogenesis for OA, we attempted to find differentially expressed genes (DEGs) in OA by obtaining GEO datasets for integrated bioinformatics analysis. Then, we performed function enrichment analysis and PPI interaction analysis to reveal the key pathways of the DEGs. In addition, the LASSO method was used to identify the key cell immune subpopulations and key biomarkers for OA. Furthermore, single-cell dataset was performed to explore the relationship between different cells in the microenvironment and identify the possible key regulatory molecules. Thus, the study aimed to identify the key molecular and cellular events involved and the immune infiltration mechanism within the synovium of patients with OA, to provide potential novel diagnostic and therapeutic targets.

## 2 Materials and methods

### 2.1 Data information

Datasets GSE1919, GSE55235, and GSE32317 are based on the Affymetrix Human Genome Array Platform. Dataset GSE46750 is based on the Illumima Genome Human Array Platform. Datasets GSE89408 and GSE143514 are based on the Illumima HiSeq RNA sequencing data, and dataset GSE152805 is based on single-cell 10× Genomics sequencing. All of the above datasets were downloaded from Gene Expression Omnibus (GEO) database (https://www.ncbi.nlm.nih.gov/geo/). All selected datasets were genome-wide expression data of OA or normal synovial membrane tissues. We obtained 5, 10, 10, and 12 normal samples and 5, 10, 9, and 12 OA samples from the array datasets GSE1919, GSE55235, GSE32317, and GSE55235, respectively. In addition, 28 and 3 normal samples and 22 and 5 OA samples were obtained from the RNA-seq dataset GSE89408 and GSE143514, and obtained 3 synovial samples of OA in GSE152805 single-cell dataset. The normal samples used in the study were all synovial tissues from accidental death or post-traumatic joint surgery or traumatic joint injury, whereas the samples in the OA group were synovial samples from patients diagnosed with synovitis who underwent open synovectomy and joint replacement. In total, we obtained 37 normal healthy samples and 36 OA patient samples from Array dataset, 31 normal healthy samples and 27 OA patient samples from RNA-seq dataset, and 3 OA patient samples from 10× Genomics single-cell sequencing dataset (**Table S1**). Due to the different sources of all datasets, we only used the Array dataset for the differential expression analysis of the most critical biomarkers in OA, used the RNA-seq dataset to verify whether the expression of biomarkers, and used the single cell sequencing dataset for further explore the conclusion based on the results.

### 2.2 Differentially expressed and gene screening

The limma package (RMA algorithm) was used to identify the DEGs between OA synovial membranes and normal group in the GSE1919 and GSE55235 datasets (26). After background adjustment, normalization, and summarization preprocessing, *P*-values were corrected using the Benjamini and Hochberg test. GSE89408 and GSE143514 gene raw read counts were used to perform different analyses with DESeq2 (v 1.18.1), which is an R package that uses a model based on the negative binomial distribution and which is widely used for RNA-seq data differential analysis (27). All the analyses results were performed by volcano plot and heatmap plot to show the DEGs, respectively. DEGs with *P* < 0.05 and |Log_2_FC| > 1 were considered as the cutoff criterion.

### 2.3 Gene ontology and pathway enrichment analysis

To investigate key mRNAs at molecular and functional levels, Gene Ontology (GO) and Kyoto Encyclopedia of Genes and Genomes (KEGG) pathway functional enrichment analyses were performed. GO analysis includes the categories of molecular function (MF), cellular component (CC), and biological processes (BPs). Pathway analysis is the process of classifying large genes by the KEGG database. In our study, GOseq uses the Wallenius non-central hypergeometric distribution model, thus taking gene length bias into account; therefore, it was used to perform GO enrichment analysis and GO terms with *P* < 0.05 were considered significantly enriched (28). KEGG Orthology Based Annotation System (KOBAS 3.0) (29) software was used to test statistical enrichment in KEGG pathways, and pathways with a Fisher’s exact test *P* < 0.05 were considered significantly enriched. Furthermore, we used the Metascape database (https://metascape.org/) to perform functional cluster enrichment analysis, which is a web-based portal designed to provide a comprehensive gene list annotation and analysis resource for experimental biologists. Enriched GO-based bp terms and pathways were considered statistically significant when the *P*-value was < 0.05.

### 2.4 Protein–protein interaction network construction and module analysis

Search Tool for the Retrieval of Interacting Genes/Proteins (STRING) (https://string-db.org/cgi/input.pl) (30) is an online database resource search tool for the retrieval of interacting genes, which includes both physical and functional associations. In this paper, the cytoscape StringsApp package was used to construct a PPI network of candidate DEGs gene sets, with a confidence score > 0.4 defined as significant (31). Then, the cytoscape ClueGo package was used to analyze the GO and KEGG pathway networks (32). Based on the above data, Molecular Complex Detection (MCODE) (33) with default parameter was then performed to monitor PPI network modules (34), then the core module analyzed by Mcode was subset to new network, and the functionally similar network genes were retained use cytoscape software version 3.7.2.

### 2.5 Immune infiltration analysis

To determine OA synovial membrane invasion from the expression data, normalized gene expression data were used to infer the relative proportions of several types of infiltrating immune cells using xcell method as previously reported (35). Briefly, gene expression datasets were prepared using standard annotation files and analyzed using the immunedeconv package, then the xcell algorithm was used and the proportion of 36 immune cells was obtained; the relative percentage of each kind of immune cell in the samples was calculated. To determine the difference in immune cell infiltration between the synovial tissue of patients with OA and that of normal controls, all immune cell proportions and ratios were compared using a non-parametric Wilcoxon rank-sum test with Benjamini–Hochberg corrections and a *P*-value threshold of 0.05 for statistical significance. The “ggplot2” package was used to draw box diagrams to visualize the differences in immune cell infiltration.

### 2.6 Gene set variation enrichment analysis

Gene set variation analysis (GSVA) can estimate the relative enrichment of a gene set of interest over a sample population, which is used to observe the variation in the activity of a set of genes corresponding to a particular biological condition [21]. As previously described, The GSVA R package (36) was used to analyze the pathway pattern between two different groups of samples, and the defined gene set was downloaded from the Molecular Signature Database, named “h.all.v7.2.symbols.gmt” (37). In the calculation, we used “zscore” algorithm to give comprehensive score to each sample and used this enrichment score to represent the degree of absolute enrichment of a gene set pathway, while different scores also represent the activity degree of the pathway in the sample. We demonstrated the correlation between pathway GSVA score and immune cells infiltration by calculating the Person correlation coefficients, which was performed by using R software. The *P*-value obtained from the correlation calculations was corrected by using the BH method, and the degree of correlations was shown by heat maps, where the asterisks were used to indicate the significance.

### 2.7 Identification of the optimal diagnostic gene biomarkers

To identify the optimal diagnostic gene biomarkers for OA inflammatory response, we utilized the immune infiltrating cell types as the biomarkers to distinguish and predict the OA inflammatory responses. Six differential percentages of immune infiltrating cells obtained from all the Array datasets were used as feature variables to construct the least absolute shrinkage and selection operator (LASSO) model. The LASSO algorithm analysis was performed by the “glmnet” R software package to achieve our datasets shrinkage. The optimal value of λ was determined by 10-fold cross-validation, and the significant variables were selected for risk prediction. The coefficient risk score of each sample was obtained according to the proportion of each significant difference immune-infiltrating cell types and their correlation. The coefficient risk score was calculated as follows: ∑*n* = *n* (Coef.k×Cell.Prop.k), the Cell.Prop.k was the relative infiltrating proportion of the infiltrating cell type of the patient k, and Coef.k indicated the LASSO coefficient of gene k. Then, all the samples were randomly assigned to the training set (70%) and test set (30%). To evaluate the diagnostic ability of the above models, we evaluated the receiver operating characteristic (ROC) area under the curve (AUC), sensitivity, and specificity. In addition, two OA synovial inflammation RNA-seq datasets from GEO database were used to validate the sensitivity and specificity of our model.

### 2.8 Single-cell analysis

The single-cell raw matrix data from GSE152805 were downloaded and imported using the Seurat package for the R programming language (version 4.0.2) (38), and the data were filtered to include genes detected in > 5 cells. Cells with 1,000–5,000 detected genes and a unique molecular identifier of 1,000–30,000 and < 10% being mitochondrial genes. After data normalization, highly variable genes of the single cells were identified after controlling for the relationship between average expression and dispersion. Then, principal components analysis (PCA) was performed, and significant principle components (PCs) were used as input for graph-based clustering. For clustering, we used the function FindClusters that implement the shared nearest neighbor (SNN) modularity optimization-based clustering algorithm on 40 PCA components with resolutions of 0.1–0.5, leading to five to 12 clusters. To be consistent with the originally published paper, a resolution of 0.2 was chosen for further analysis. To identify DEGs in each cell cluster, we applied the FindAllMarkers function with the default parameter from Seurat set to the normalized gene expression data. Subsequently, the cell clusters were identified by the cell type–specific biomarkers and the proportions of the cell types were calculated and evaluated.

### 2.9 Cell communications analysis and Ligand–Receptor expression

Cell–cell communications (CCCs) analysis assesses the expression of ligand-receptor pairs across cell types and revealed specific signaling pathways (39). CellChat analysis uses to reveal the afferent communication patterns and secreted efferent communication patterns of each cell type, to quantify the cell communication Pathway, and to calculate the information flow of each signal Pathway or the communication probability across the cells (40). In our study, CellChat was used to analyze the OA synovial arthritis single-cell samples. By performing CellChat, we calculated and analyzed the intercellular communication of cell types of each OA inflammation sample by importing the standardized scRNA-seq data after Seurat package analysis into CellChat. Among the OA inflammation cellular communication signals, we conducted in-depth analysis on Macrophage to reveal the communication strength of each signaling pathway and selected the specific communication pathways for further visualization. The default parameters of the software were used in our CellChat analyses, and *P* ≤ 0.05 was used as a threshold for significance, and Adjust *P*-value was corrected using the BH method.

### 2.10 Scissor analysis

Single-Cell Identification of Subpopulations with bulk Sample phenotype correlation (Scissor) uses leveraging bulk data and phenotype information to identify biologically and clinically relevant cell subsets form single-cell RNA sequencing, with high accuracy and specificity (41). The cell types form single-cell sequencing samples typically includes normal cells and the disease associated cells. Scissor uses to identify the disease relevant subpopulations form single-cell sequencing samples by using phenotype optimization correlation matrix regression model. In our study, we used the normalized expression profile date of GSE1919 and GSE55235 combined with the clinical phenotype information of each sample in the dataset to analyze the Seurat expression profile of single-cell sequencing dataset GSE152805, and to obtain the most relevant OA synovial inflammation cell types form single-cell sequencing samples. Then, we used Scissor analysis to further explore and analyze the mechanism of disease pathogenesis and progression for Macrophage types. In Scissor analysis results, the background cells and negatively correlated with the phenotype cells were combined and labeled Scissor0; the cell types positively correlated with the phenotype were labeled Scissor1.

### 2.11 Statistical analysis

All the statistical analyses were executed with R software (version 4.0.2). *P* values were calculated using Wilcox.test, and *P* < 0.05 was considered statistically significant.

## 3 Results

### 3.1 Identification of DEGs and functional analysis shows OA involved in immune-related pathways

**Figure 1:**
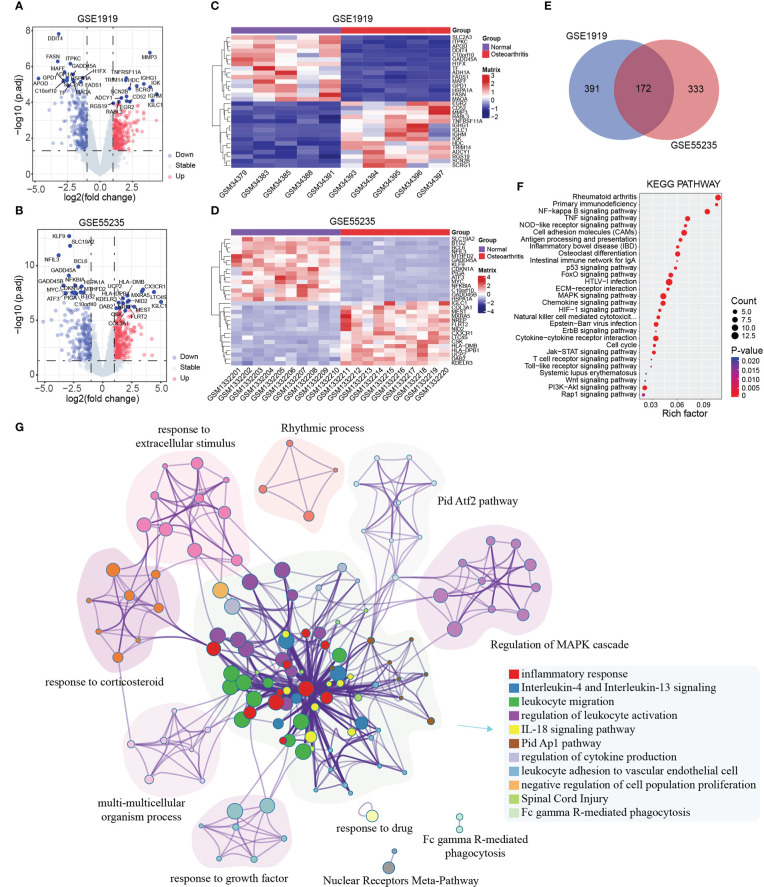
Differential expression genes and function analysis. **(A, B)** Volcano plot showed DEGs in GSE1919 and GSE55235, respectively. Blue dots represented downregulated DEGs, red dots represented upregulated DEGs, and gray dots represented the rest of the no significant differential expressed. **(C, D)** Heatmap showed the potential top 15 DEGs in GSE1919 and GSE55235, respectively. **(E)** Venn plot of the common DEGs of GSE1919 and GSE55235. **(F)** KEGG function analysis of the 172 DEGs. **(G)** Metascape function clustering analysis of the 172 DEGs.

To identify the DEGs in OA, we downloaded the OA synovial and normal tissue gene expression profiles of GSE1919 and GSE55235 from the GEO database. Then, we performed differential analysis, a total of 563 DEGs were identified in GSE1919 dataset, including 293 upregulated and 270 downregulated DEGs, and a total of 505 DEGs were identified in GSE55235 dataset, including 287 upregulated and 218 downregulated DEGs (**Figures 1A, B**). Heatmap Cluster analysis showed the potential top 15 DEGs between the OA synovial membrane and normal tissues (**Figures 1C, D**). To identify the common DEGs of important inflammatory regulatory factors in OA synovitis, we performed an integrative analysis, a total of 172 common DEGs overlapped in GSE1919 and GSE55235 datasets (**Figure 1E**; **Table S2**). Furthermore, we analyzed the functions by using GO and KEGG enrichment analysis with the 172 common DEGs. GO enrichment analysis results showed that the 172 common DEGs were mainly involved in the BPs of immune system, inflammatory and development, the cellular sub-localization of extracellular matrix and vesicles, and the molecular biological functions of oxidase, receptor activity, and kinase binding (**Supplementary Figures S1A–C**). KEGG enrichment analysis results showed that the 172 common DEGs were mainly involved in immune-related pathways, such as Rheumatoid arthritis, NF-kappaB signaling pathway, Osteoclast differentiation, TNF signaling pathway, MAPK signaling pathway, and T-cell receptor signaling pathway (**Figure 1F**; **Table S3**). These results were consistent with the previous results and demonstrated the aberrant expression of these genes lead to inflammatory response in OA synovitis tissues (42, 43). On the other hands, we performed the clustering analysis of these DEGs for further clearly specific function using Metascape software (**Figure 1G**), and these genes mainly enriched and clustered eight categories, including inflammatory response, regulation of MAPK cascade, Pid Atf2 pathway, Thythmic process, response to extracellular stimulus, response to corticosteroid, multicellular organism process, and response to growth factor.

### 3.2 Protein–protein interaction analysis of the differentially expressed genes

**Figure 2:**
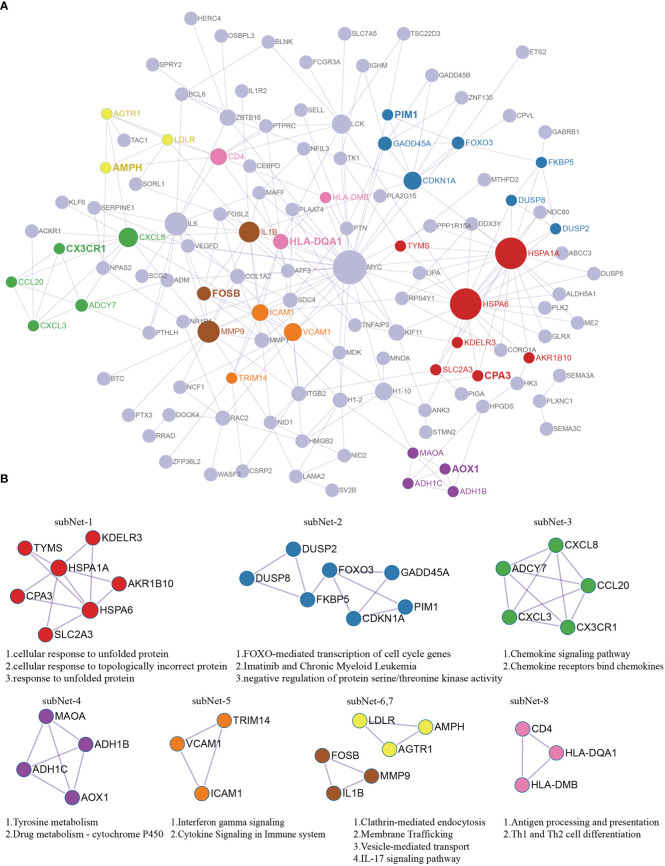
Protein–protein interactions analysis with 172 DEGs. **(A)** PPI network of DEGs. Red nodes labeled subNet1 indicate the cellular response of unfolded proteins; blue nodes labeled subNet2 indicate the regulation of cell cycle and kinase activity; green nodes labeled subnet3 indicate chemokine signaling pathway; purple nodes labeled subnet4 indicate the metabolic processes; orange nodes labeled subnet5 indicate the immune system signaling; yellow nodes and brown nodes labeled subnet6 and subnet7 indicate the signal translocation and inflammation during biofilm processes; pink nodes labeled subnet8 indicate antigen the presentation and T-cell differentiation. **(B)** The key subnetworks screened after using MCODE tool.

To further improve the biological understanding of the correlation between the 172 DEGs gene functions identified in our study, we next conducted the protein–protein interaction analysis. The STRING database was used to identify the PPI network analysis of 172 DEGs; the results showed that 121 nodes and 257 edges were established in the constructed PPI network and visualized by Cytoscape software (**Figure 2A**). These genes include HSPA1A (HSP70), HSPA6, MYC, IL6, MMP9, CXCL8, LCK, and VCAM1, which are most edges connections, suggesting that these genes may act as an important role in the process of inflammation. To further identify the subnetworks of involved inflammatory reaction, we firstly performed functional analysis to further identify the pathways and functions of this PPI network by using ClueGo tool. Then, MCODE tool was used for further subnetwork analysis. Finally, eight key subnetworks (subnet1-8) with independent biological functions were extracted (**Figure 2B**). Subnet-1 was mainly involved in stress-apoptotic signaling pathways such as cellular response of unfolded proteins; subnet-2 was involved in proliferation signaling pathways such as the regulation of cell cycle and kinase activity, FOXO-mediated transcription of cell cycle genes; subnet-3 was mainly involved in inflammation-related signaling pathways such as chemokine signaling pathway; subnet-4 was involved in metabolic pathways such as Tyrosine metabolism; subnet-5 was involved in immune system signaling; and subnet-6 and 7 were involved in signal translocation and inflammation during biofilm processes, whereas subnet-8 was involved in antigen presentation and T-cell differentiation, respectively. All the related genes and their functions in the above subnetworks are closely related to the occurrence of inflammatory response and the regulation of microenvironment, which may play an important role in the progression of OA disease.

### 3.3 Identification of immune cell types with OA based on immune infiltration

**Figure 3:**
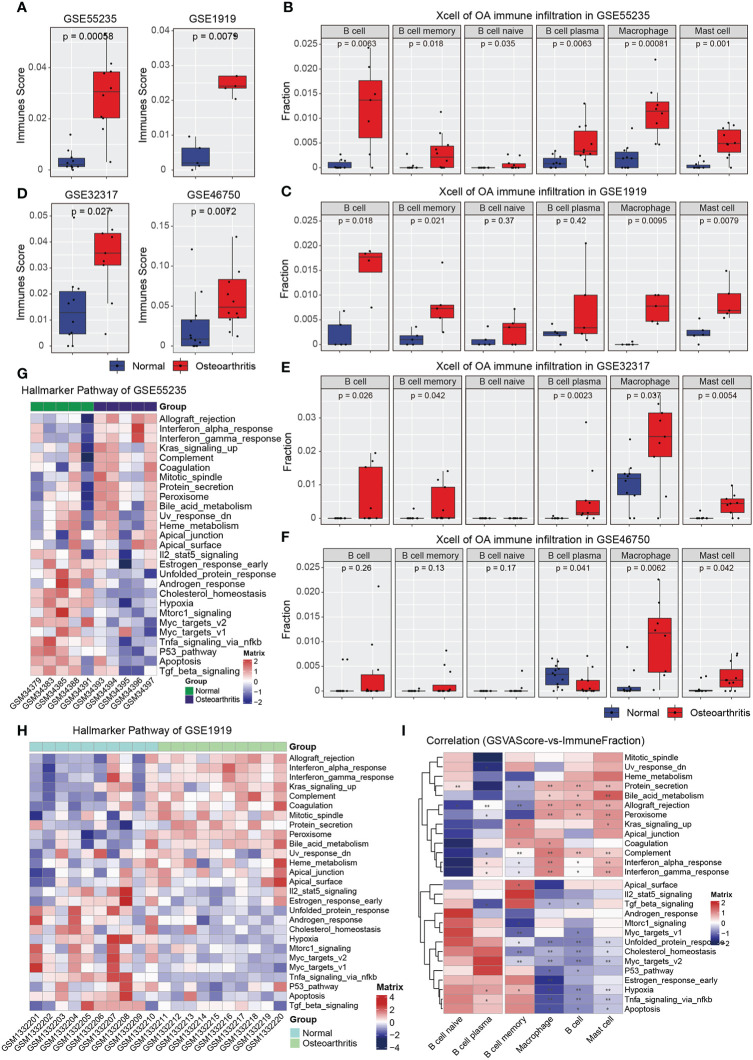
Immune infiltration analysis and GSVA analysis of OA samples. **(A)** The immune score of GSE55235 and GSE1919 samples. **(B)** The immune infiltration of GSE55235 samples. **(C)** The immune infiltration of GSE1919 samples. **(D)** The immune score of GSE32317 and GSE46750 samples. **(E)** The immune infiltration of GSE32317 samples. **(F)** The immune infiltration of GSE46750 samples. **(G)** Heatmap of Hallmark gsva scores in GSE55235 dataset. **(H)** Heatmap of Hallmark gsva scores in GSE1919 dataset. **(I)** Heatmap showed that the spearman correlation between gsva score and immune infiltration cell types. (*p < 0.05; **p < 0.01).

The above results indicate that immune inflammatory associated pathways played the vital roles in the pathological process of OA. It is speculated that OA specimen may induce different immune inflammatory cell infiltration under the inflammatory environment. To confirm our hypothesis, we used the xcell method to calculate the Immunes Score in GSE1919 and GSE55235 datasets, and the results showed that Immunes Score in OA samples were consistently significantly higher than those of normal tissues in these two datasets (**Figure 3A**). Then, xcell method was also used to perform the immune infiltration analysis in the two datasets, and the results showed that the proportion of immune infiltration of six immune cell types, including B cell, B cell memory, B cell naïve, B cell plasma, Macrophage, Mast cell, were consistently significantly changed and increased in OA inflammatory samples (**Figures 3B, C**).

To obtain optimal immune infiltration for OA, we also downloaded the GSE32317 and GSE46750 datasets from GEO database to perform the xcell method to calculate the Immune score and immuno-infiltration analysis. The results showed that the Immune score were consistently significantly increased in OA inflammatory samples with the two GEO datasets above (**Figure 3D**). Additionally, the immune infiltration of five immune cell types in GSE32317, including B cell, B cell memory, B cell plasma, Macrophage, Mast cell, were significantly increased in OA inflammatory samples (**Figure 3E**), and only three immune cell types in GSE46750, including B cell plasma, Macrophage, Mast cell were significantly increased (**Figure 3F**). Overall, the immune infiltration analysis results showed that the six immune infiltration cells were largely related to the synovial microenvironment of OA.

In additional, the pathway activity of Hallmark gene sets in GSE55235 and GSE1919 datasets was further obtained by gsva enrichment analysis, which contains immunological, inflammatory, metabolic, anaerobic, and other related pathways, reflecting the degree of variation of each sample. The results showed that the inflammatory, metabolic, and stress-related pathways such as interferon, complement, UV, and metabolism were activated, whereas the anaerobic, apoptosis, and immune-related signaling pathways such as hypoxia, apoptosis, and TGFβ were inhibited in OA inflammatory samples (**Figures 3G, H**). All the consistently significantly changed signaling pathways were indicated that the inflammatory microenvironment may be closely associate to OA synovial inflammatory. Finally, the Spearman correlation analysis was used to evaluate the immune infiltrating cell types of microenvironment changes between immune infiltration Score and GSVA Score. The correlation results between six immune inflammatory cell types and related hallmark pathways were indicated that B cell memory, Macrophage, B cell, and Mast cell were significantly correlated with the GSEA pathway (**Figure 3I**). It is speculated that the infiltration degree of the four cell types may be related to the occurrence and development of inflammation in OA. These findings indicated that the infiltration degree of these four cell types might synergize with the occurrence and development of inflammation in OA.

### 3.4 Identification of the optimal diagnostic immune infiltration biomarkers for OA

**Figure 4:**
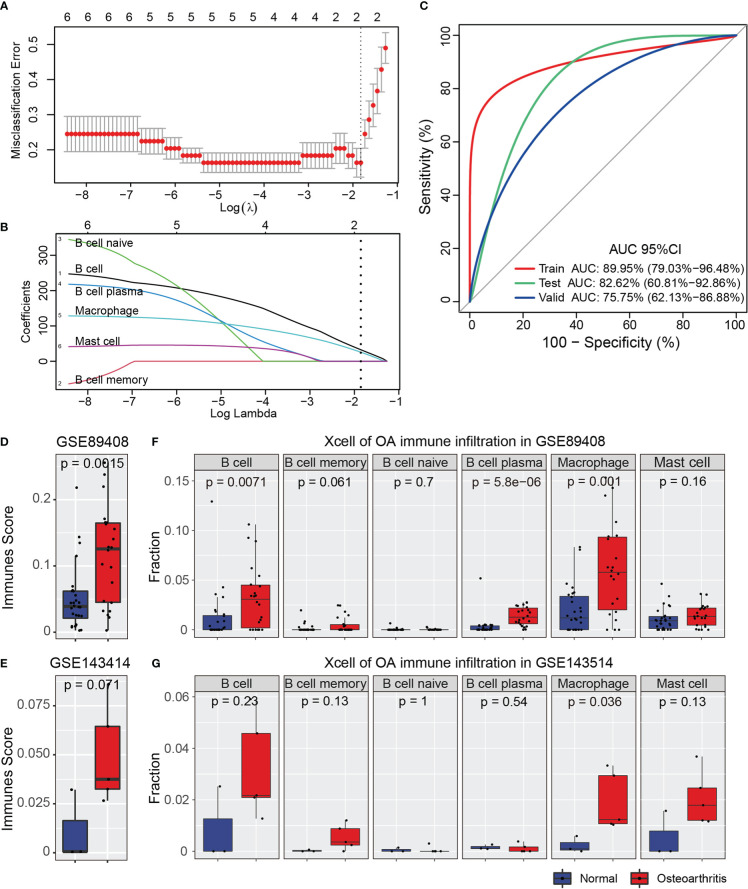
LASSO analysis for immune infiltration cell types. **(A)** CV statistical graph during the construction of the LASSO regression model, which shows that the minimum lambda at model construction is 0.1614863. **(B)** The model regression coefficient diagram shows the change trend of the coefficient corresponding to each immune infiltrating cells variable with the change of lambda value. **(C)** ROC curve predicts the identification effect of the above models in different datasets. The closer AUC value is to 1, the better of prediction effect on the model. The figure is shown that the AUC in the training set is 0.8995 and that, in the test, set is 0.8262, indicating that the model has a robust prediction accuracy. In the validation datasets, the AUC is 0.7575, which shows that the models constructed by the Macrophage and B cell can also have good accuracy in different types of datasets. **(D, E)** The immune score of GSE89408 and GSE143514 datasets. The figure shows that immune sore is significantly increased in the two datasets. **(F, G)** The immune infiltration analysis of six cell types in GSE89408 and GSE143514 datasets.

The results of immune infiltration analysis showed that the above six cell types had significant changes in OA, and we speculated that the immune infiltrative cell types could be used as biomarkers for the diagnosis and prediction for OA. To identify the optimal diagnostic cell type biomarkers for OA, we utilized the infiltration score in six immune infiltrating cells obtained from all the Array datasets to construct LASSO model (**Figure 4A**). We first conducted the LASSO regression analysis for all the samples in GSE1919, GSE55235, GSE32317, and GSE46750 datasets, which were randomly assigned to the training set (70%) and test set (30%). Then, the optimal lambda value was determined by 10-fold cross-validation. The lambda Min value was 0.1614863 (**Figure 4B**). Two significant infiltrating cell types included B cell and Macrophage cell, which were further used as a diagnostic marker, and the formula was calculated as follows: ∑*n* = B cell*30.8727 + Macrophage*24.1093. Additionally, the ROC curve analysis showed that the AUC value of the Lasso model of two infiltrating cells on the training set was 0.8995 (95% CI: 79.03–96.48%), and the AUC value on the test set was 0.8262 (95% CI: 60.81–92.86%), the model had high AUC values in both the training set and the test set, indicating that the model has a robust sensitivity and specificity, and could be used as an optimal biomarker of OA (**Figure 4C**). On the other hand, two OA synovial inflammation RNA-seq datasets of GSE89408 and GSE143514 from GEO database were downloaded to validate and verify the above LASSO model, and further to evaluate the specificity, sensitivity, and accuracy of our model. The finally validated AUC value was 0.7575 (95% CI: 62.13–86.88%), which also had a high accuracy in predicting OA.

Additionally, we then used the xcell tool to perform the immune scoring and immune infiltration analysis of GSE89408 and GSE143514 datasets, and we found that the immune score of the OA synovial inflammatory was significantly higher than normal synovial tissue (**Figures 4D, E**). The immune infiltration of Macrophage in the two datasets were significantly increased in OA inflammatory samples, and the immune infiltration of B cell in GSE89408 was also significantly upregulated, whereas in GSE143514 was maintained a consistent upward trend but without significant change (**Figures 4F, G**). Combined with the above results obtained in our study, it was suggested that the two immune infiltrating cells (Macrophage and B cell) of LASSO models could be used as diagnostic biomarkers of OA, especially Macrophage.

### 3.5 Cellular communication patterns of Macrophages in OA synovitis microenvironment

Previous immune infiltration and GSVA analysis showed that Macrophage was significantly upregulated and largely correlated to the signal pathways of microenvironment in the four GEO datasets and two RNA-seq GEO datasets of OA, so Macrophage may play an important role in OA synovial inflammation and may serve as key therapy target. To clarify the role of Macrophage in synovial inflammation of OA, we downloaded another single-cell sequencing dataset GSE152805, which included three OA synovial samples, to detailedly investigate the function and role of Macrophage. After downloading the expression matrix, three synovial samples were processed by quality filtering, PCA reduction and UMAP clustering, and finally obtained 10 cell types by cell identification (**Supplementary Figure S2A**). These cell types were defined as synovial subintimal fibroblasts (SSF) (CXCL12+), synovial intimal fibroblasts (SIF) (PRG4+), Macrophage (CD163+, LYVE1+), DC (FCER1A+, IL1R2+), endothelial cell (EC) (PLVAP+), smooth muscle cell (SMC) (RGS5+), Mast Cell (TPSAB1+), proliferating immune cell (ProIC) (CENPF+), T cell (CXCR4+), and B cell (MZB1+) (**Supplementary Figure S2E**). Subsequently, we evaluated the proportion of each subtype cells, and found that Macrophage accounted for 5.09–31.63% in the three samples (**Supplementary Figure S2B**). Based on the cell annotation, we identified the differently DEGs by each cluster and showed by Heatmap and dotplot (**Figures S2C, D**).

**Figure 5:**
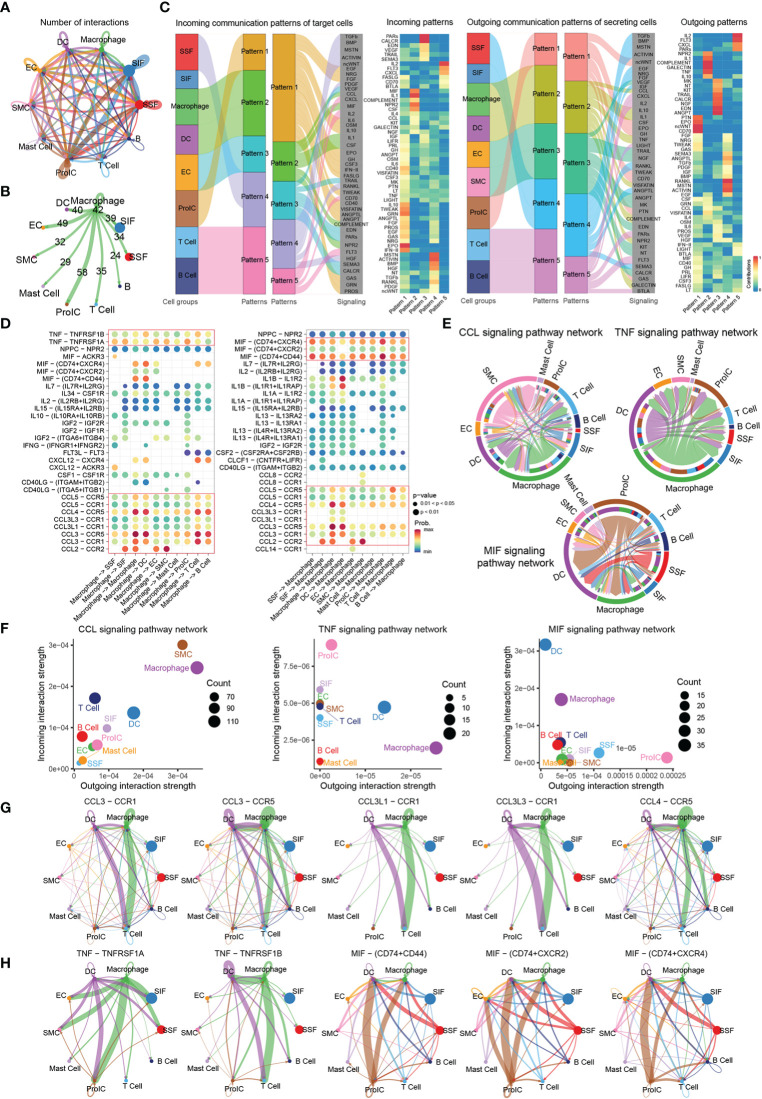
Cellular communication of Macrophage in OA. **(A)** Circle plot of the significant connections among ten interacting immune cell types (Macrophage, DC, EC, SMC, Mast cell, SIF, SSF, ProIC, T cell, and B cell). Different colors represent different cell groups. **(B)** Circle plot of the significant connections of Macrophage with other cells. **(C)** Pattern recognition of the immune cell types; the graph shows the interpretation of intercellular communication networks by incoming and outcoming communication patterns. **(D)** Bubble plot of the ligand-receptor pairs in Macrophage cell types. Colors in the bubble plot are the proportional of the communication probability, where the blue and red colors correspond to the smallest and largest values. **(E)** Circular plots of the communication among the 10 immune cell subtypes according to the three major signaling pathways (CCL, TNF, and MIF). Different colors represent different immune cell types. **(F)** Cellular communication strength on a two-dimensional manifold according to incoming and outcoming communication patterns; each dot represents one cell type. **(G, H)** Communication probability by ligand-receptor pairs according to CCL, TNF, and MIF signaling pathway, each dot represents the communication network of one immune cell types. Line size is proportional to the overall communication probability.

To exploring detailed regulations of Macrophage in the development of inflammation in OA synovial microenvironment, CellChat analysis was performed to infer, visualize, and analyze inter-cellular communications form scRNA-seq data. Significant connections among 10 interacting immune cell types were identified. Notably, several cell types such as SIF, SSF, and ProIC were found to have more interacting cell communication pairs in the OA synovial microenvironment (**Figure 5A**). Importantly, CellChat also predicted that Macrophage had connection with the other immune cell types, with different cell communication pairs (**Figure 5B**). On the other hand, we also identify the cell communication patterns, including outgoing patterns and incoming patterns; the results showed that five incoming communication patterns were identified, and Macrophage communicate with other cell types in incoming signaling was dominated by pattern 2, which include signaling pathway such as CCL, MIF, IL1, CSF, Complement, and NPR2 whereas, in five outgoing signaling patterns, Macrophage communicate with other cell types was characterized by pattern 2, which include signaling pathway such as CCL, IL10, IL1, TNF, VISFATIN, COMPLEMENT, NPR2, and GALECTIN (**Figure 5C**). Furthermore, we detected the significant ligand-receptor pairs between Macrophage and the other cell types, which were further categorized into two communication patterns. The outgoing communication ligand-receptor pairs were characterized by CCL and TNF signaling pathway, whereas the incoming communication ligand-receptor pairs was characterized by CCL and MIF signaling pathway of ligand-receptor pairs (**Figure 5D**). To further investigate the above three signaling pathways (CCL, TNF, and MIF) in OA synovial microenvironment, we performed chord analysis of the three signaling pathways and found that CCL signaling pathway was mainly used by SMC and Macrophage cells for incoming and outgoing communication, TNF signaling pathway was mainly used by Macrophage and DC cells for outgoing communication, and MIF signaling pathway was mainly used by DC, ProIC, and Macrophage cells for incoming communication (**Figure 5E**). Finally, the communication intensity analysis could be also found that CCL signaling pathway was consistently used in incoming and outgoing communication, whereas TNF signaling pathway was mainly used in Outgoing communication and MIF signaling pathway was mainly used in Incoming communication (**Figure 5F**). These results showed that Macrophage mainly uses CCL, TNF, and MIF signaling pathways for cell communication in the OA synovial microenvironment.

Based on the above three signaling pathways (CCL, TNF, and MIF), we further analyzed the communication of ligand-receptor pairs. Notably, five ligand-receptor pairs including CCL3-CCR1, CCL3-CCR5, CCL3L1-CCR1, CCL3L3-CCR1, and CCL4-CCR5, were the dominant contributors to CCL signaling pathways, which has the strongest communication between macrophage and T cells, and this result suggested that Macrophage uses this signaling pathway to recruit T cells in the microenvironment to cope with the occurrence and development of inflammation (**Figure 5G**). TNF signaling pathways mainly consist of TNF-TNFRSF1A and TNF-TNFRSF1B ligand-receptor pairs, which was mainly used in Outgoing communication between Macrophage and ProIC/T Cells, and the result suggested that Macrophage also uses this signaling pathway to recruit immune cells in response to changes in the microenvironment (**Figure 5H**). Similarly, three ligand-receptor pairs of MIF signaling pathways included MIF-CD74-CD44, MIF-CD74-CXCR2, and MIF-CD74-CXCR4, which was the strongest communication between Macrophage and ProIC/DC, and this result suggested that the proliferative immune cells are responding to changes such as microenvironment inflammation (**Figure 5H**).

Taken together, CellChat identify key features of Macrophage communications within the microenvironment of OA synovial inflammation and predict that CCL, MIF, and TNF signaling pathways are the mainly cellular communication pathways and Macrophage may be used as the future therapeutic target.

### 3.6 Identifying M2-like macrophage related to anti-inflammation of OA

**Figure 6:**
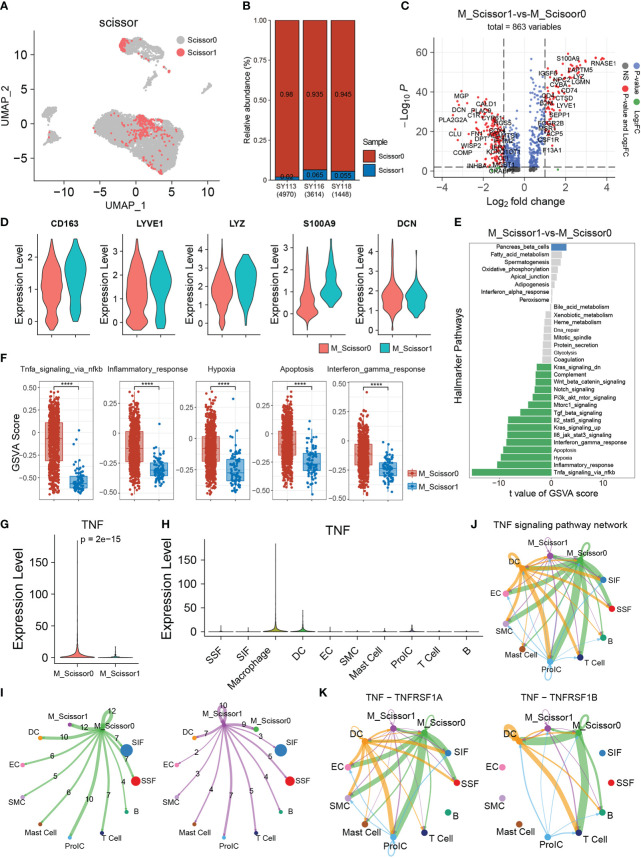
Scissor identification results on Macrophage. **(A)** The UMAP visualization of scRNA-seq datasets, scisoor1 represents that cell identified to be positively associated with transcriptome synovial inflammation, scissor0 were the background cells. **(B)** The bar plot shows the proportion of scissor cells in the three scRNA-seq samples. **(C)** The volcano plot of differential gene expression in scissor1 Macrophages (M_Scissor1) *versus* scissor0 Macrophages (M_Scissor0). **(D)** The violin plots show the several significant upregulated genes (RNASE1, CQB, S100A9, LYZ, and CD163) in M_Scissor1 group. **(E)** GSVA enrichment analysis of the hallmarker signaling pathways in Macrophage scissor group. **(F)** The Box plot shows the significant signaling pathways in gsva analysis. **(G)** The TNF gene expression in Macrophage scissor group and specific express in M_Scissor1 group. **(H)** The violin plot showing TNF gene expression in each cell type. **(I)** The cell communication number in Macrophage scissor group cells. **(J)** TNF signaling pathway usage in each cell clusters. **(K)** The ligand-receptor pairs usage of TNF signaling pathway in each cell clusters.

To utilize phenotype information of Array datasets, we performed Scissor to identify cell subpopulations in single-cell dataset that are most highly associated with the OA progression. These results indicated that the cells, which most relevant with OA synovial inflammatory were labeled as Scissor1, mainly distributed in SIF, Macrophage, and ProIC cell clusters (**Figure 6A**; **Supplementary Figure S2A**). In contrast with Scissor0 (background cells), the proportion of Scissor1 cell which identified in the three scRNA-seq samples were 2–6.5% (**Figure 6B**). Interestingly, 9.35% of Macrophage cells were identified to be most associated with the phenotype in OA.

To further understand the function of the Macrophages labeled with Scissor1 (M_Scissor1), we performed differentially expressed analysis between these cells and other Macrophage cells (M_Scissor0). As a result, 181 upregulated genes such as CD163, LYVE1, LYZ, S100A9, CD74, CD4, SEPP1, C1QB, and RNASE1 were significantly expressed in Scissor1 Macrophage group, whereas 287 downregulated genes such as MGP, CALD1, DCN, CLU, and SOX4 were significantly expressed in Scissor0 Macrophage group (|Foldchange|≥1.5) (**Figure 6C**). Several significant differently expressional genes were visualized by violin plot, which were confirmed the accuracy of these differently expressional genes (**Figure 6D**). Interestingly, we found LYVE1 and CD163 in the upregulated genes, which are also markers of M2 Macrophages, combined with the expression of CD163 and LYVE1 (**Supplementary Figure S2E**); we suggested that the identified Macrophages, which labeled scissor1, were a group of anti-inflammatory M2-like Macrophages. Next, we performed GSVA enrichment analysis in Scissor1 Macrophages and Scissor0 Macrophages to explore the activity of inflammation-related signaling pathways; the result found that multiple inflammation-related signaling pathways associated with Scissor1 Macrophages were in a low activity state, including signaling pathways that promote inflammation, such as TNF signaling, Inflammatory response, Hypoxia, Apoptosis, and Interferon pathways (**Figures 6E, F**). These results were consistent with our suggestions that Scissor1 Macrophages is a group of M2-like Macrophages with more anti-inflammatory ability.

Combined with the results of cell communication and Scissor analysis, we hypothesized that TNF signaling pathway is a key signaling pathway for cell communication in OA synovial inflammatory microenvironment. To prove our hypothesis, we detect the TNF gene expression in each cell type and compared the expression of TNF gene between Macrophages which labeled M_Scissor1 and M_Scissor0; we found that TNF gene was specific express in Macrophages and significantly down-expression in Scissor1 Macrophages cells (**Figures 6G, H**). Similarly, we performed CellChat analysis and compared the cell communication ability between Scissor0 Macrophages and Scissor1 Macrophages, the result showed that the Scissor1 Macrophages cell communication number was lower than Scissor0 Macrophages (**Figure 6I**). Also, we analyzed the cell communication network of TNF pathway in each cell type; the result showed that there were low communication levels of TNF pathway in Scissor1 Macrophages, whereas there was a high level of cell communication of TNF pathway in Scissor0 Macrophages (**Figure 6J**), which was consistent with our speculation. The TNF pathway is a key active signaling pathway in OA synovial arthritis microenvironment. Finally, we analyzed ligand receptor use in the TNF pathway and found that all ligand receptor communication was highly used only in Scissor0 Macrophages, whereas Scissor1 Macrophages maintained a low level of communication (**Figure 6K**).

Collectively, these results suggests that Macrophages are an important biomarker and be a potential therapeutic target in OA synovitis, in which TNF signaling pathways may play a critical role in the pathogenesis of OA.

## 4 Discussion

OA is a progressive joint disease that is found worldwide; it influences the whole joint, including tissue such as cartilage, subchondral bone, and the synovium (44). Recent research has shown that synovitis is one of the most common characteristics of OA from the early stage to the late stage. This results in bone and cartilage damage through the formation of the inflammatory pannus; furthermore, it plays an important role in the progression and pathogenesis of OA (45, 46). Therefore, understanding the molecular mechanism of synovitis in OA is necessary. In this study, we first comprehensively analyzed two datasets, GSE1919 and GSE55235, from the NCBI-GEO database to identify DEGs in the synovium of OA, and we successfully identified 172 DEGs that were in both datasets. Then, we performed GO enrichment analysis, KEGG pathway analysis, and clustering analysis of the DEGs to investigate their associated biological functions.

According to the GO analysis, the DEGs were mainly enriched regarding several BPs, especially immune system and inflammatory. It has been reported that the immune system process and inflammatory both play a role in OA development and progression, which is one of the key factors in the pathogenesis of OA (47, 48). Then, the enriched KEGG pathways of DEGs mainly consisted in immune-related pathways, such as TNF signaling pathway, NF-kappaB signaling pathway, MAPK signaling pathway, T-cell receptor signaling pathway. Additionally, we performed the clustering analysis of these DEGs genes for further clearly specific function, and these genes mainly enriched eight categories, including inflammatory response, regulation of MAPK cascade, PidAtf2 pathway, Thythmic process, response to extracellular stimulus, response to corticosteroid, multicellular organism process, and response to growth factor. Overall, the above results suggested that immune system and inflammatory was mainly involved in the pathophysiological processes of OA, which were consistent with previous OA studies (49–51). Previous studies have demonstrated that inflammatory reaction mediated pathophysiological processes occurring in OA, which has been proposed that targeting inflammatory could be a promising therapy (52–54).

In the PPI network analysis, the DEGs were mainly enriched in the process of pathogenesis in OA, which were identified about eight key subnetworks with the hub genes HSPA1A(HSP70), HSPA6, MYC, IL6, MMP9, CXCL8, LCK, VCAM1, the enriched function such as cellular response of unfolded proteins, cell cycle and kinase activity, chemokine signaling pathway, metabolic processes, signal translocation and inflammation during biofilm processes, and antigen presentation and T-cell differentiation. As reported in OA, HSPA1A protected against OA by inhibiting chondrocyte apoptosis, which were specific upregulated by RNA-binding protein ZFP36L1 (55). The similar conclusions were drawn in a related study on the MYC, OA-related key genes and were identified and clinical validated in OA (56). Previous evidence showed that MMP9 and CXCL8 were the novel targets for OA immunotherapy and diagnosis in the underlying biological mechanisms of OA pathogenesis (57–59). CXCL8 was reported to be elevated in the serum and synovial fluids of patients with OA; moreover, it plays a pro-inflammatory role in chondrocytes in OA (60).

Then, we performed Immune infiltration analysis on GSE55235, GSE1919, GSE32317, and GSE46750 datasets; the results showed that six immune-related cell types including B cell memory, Macrophage, B cell, B cell naïve, B cell plasma, and Mast cell were significantly correlated with the OA. These different immune cell types have been indicated that the immune infiltration degree might synergize with the occurrence and development of inflammation in OA (12, 61). Next, we considered that these immune cell types may be used as the optimal biomarkers of the diagnosis and prediction for OA. We further observed the percentages of immune infiltration of six immune cell types by LASSO analysis. Finally, we found that two immune infiltrating cells with Macrophage and B cell had significant changes and could be used as diagnostic biomarkers of OA, especially Macrophage. Recent studies have reported that Macrophage infiltration in the synovial tissue of OA is relatively high (62–64). Macrophage accumulation is the common features of OA, with different roles depending on their different phenotypes (13, 17, 65). Moreover, emerging reports reveal that pro-inflammatory (M1) Macrophage infiltration exacerbates the pathological process of OA (15). Transient receptor potential vanilloid 1 (TRPV1) could inhibit the M1 Macrophage polarization to attenuate the progression of OA by Ca2+/CaMKII/Nrf2 signaling pathway (66). Ziming Chen et al. showed activated mast cells were mainly associated with high immune cell infiltration in OA. Furthermore, they speculated that anti-inflammatory (M2) Macrophages in the synovium and mast cells in the subchondral bone may play an important role in the pathogenesis of OA (12, 14). The above evidence combined with these results show that Macrophage in the synovium may be highly related to the infiltration of OA. Even though Macrophage plays significant roles in OA, the phenotypes are attractive issues, and further studies should clarify the Macrophage phenotypes and mechanisms as the biomarkers in OA patients.

Therefore, it is necessary to explore the specific mechanisms of Macrophage in the development of inflammation in OA synovial microenvironment. We used the CellChat tool to analyze the interaction between different cell types in the OA synovial microenvironment. The results showed that Macrophages had significant interactions with SIF, SSF, and ProIC cells. Three major signaling pathways including CCL (C-C motif Chemokine ligand), TNF-α (Tumor necrosis factor α), and MIF (Macrophage migration inhibitory factor) were the mainly signal pathway of Macrophage in OA synovial microenvironment. Among these, CCL signaling pathway is reported to perform a pleiotropic effect on multiple cell type–related OA aside from chemotaxis, with the most important chemokines such as CCL2, CCL3, CCL4, and CCL5 (67, 68). Moreover, the TNF signaling pathway serves an essential role in synovial fibroblasts in OA (51). JUN, a transcription factor, plays a significant role in the TNF signaling pathway (25, 69). Ming Liu found that MIF is a procytokine that mediates pleiotropic inflammatory effects in OA patients, reducing MIF mRNA and protein expression that could play a protective role in OA (70). Thus, these results, some of which were consistent with the previous studies, further suggest that Macrophage play a critical role in OA immune regulation and responses *via* cellular communications by the major signaling pathways.

At last, we performed Scissor analysis, which uses single-cell dataset, to identify associated cells consistent with the OA phenotype and function, such as identifying a population cell with greater proinflammatory capacity in M1 macrophages. The result showed that the identified cells were mainly distributed in Macrophage, SIF, and ProIC cells. Interestingly, we found that a group of cells were also identified in macrophages, and this group of cells highly expressed biomarkers of M2 macrophages, such as CD163 and LYVE1, and we also found that these cells had anti-inflammatory ability, which was consistent with the function of M2 macrophages in OA synovitis. For example, Alpha defensin-1 promotes M2 Macrophage polarization, indirectly effect on chondrocytes to attenuate the severity of OA (71). Moreover, several phenotypes of M2-like Macrophages were identified depending on the differentiation signal known markers such as CD163, CD206, Arg1, and CCL22 (72). Finally, we explored the differences cell communication ability of these cells and found that the number of identified M2-like macrophages was slightly lower than other macrophages; however, the usage of TNF signaling pathway was significantly reduced. Combined with several reports that M2 Macrophage have the functions of promoting angiogenesis and tissue repair and TNF is a pro-inflammatory gene that specifically expressed in macrophages, we have a reason to believe that M2-like macrophages may be a candidate cell for future clinical treatment in OA, and TNF gene may be one of the key molecules.

The lack of results from own samples and the absence of experimental validation is a major limitation of this study, and the differences among individuals in biological samples may also have some influence on the results. Although four microarray data, two RNA-seq data, and one single-cell data were used in our analysis to demonstrate our point, the combination of multiple data sets only can reduce the conclusion bias generated by a single data method. Expanding the sample size and obtaining clinical sample for biological verification and exploring the specific functions of macrophages can effectively prove our conclusion.

In summary, our study combined integrated bioinformatics and machine learning methods to identify the immune regulation and immune infiltration during OA pathogenesis. The above results show that Macrophage could be a rational candidate cells for OA, and TNF gene may be a key molecule in the development of OA; however, the concrete mechanism should be elucidated in further studies.

## 5 Conclusions

In this study, a total of 172 DEGs were identified in the OA synovium, and Macrophage infiltration could be a rational biomarker. In addition, TNF may be a key molecule. However, the key genes and related mechanisms involved in immune regulation and immune infiltration in the pathogenesis of OA need to be further studied, and the function of macrophages needs to be further explored.

## Data availability statement

The original contributions presented in the study are included in the article/**Supplementary Material**. Further inquiries can be directed to the corresponding authors.

## Author contributions

GN and XF conceived and designed the experiments. SL, MY, YeW performed the experiments, analyzed the data, contributed reagents and materials, prepared figures and tables, authored or reviewed drafts of the paper. XF wrote the manuscript. All authors contributed to the article and approved the submitted version.

## Supplementary material

Supplementary Figure 1 GO functional analysis 172 genes associated with OA. **(A–C)** The GO analysis result showed by biology process, cellular component, and molecular function.

Supplementary Figure 2 Single-cell analysis with three synovial samples of OA. **(A)** The UMAP showing 10 cell types were identified. **(B)** The percentage of 10 cell types in each sample. **(C)** Heatmap of single-cell data based on the UMAP plot. Columns represent individual cells and rows represent genes. **(D)** The dot plot showing the expression of marker genes in each cell type; the size of the dot reflects the percentage of cells expressing the gene; expression levels are color coded. **(E)** UMAP plot showing the marker gene expression in all cells.
