# Supplementary figures and images for: Comprehensive bulk and single-cell transcriptome profiling give useful insights into the characteristics of osteoarthritis associated synovial macrophages
Source: Front Immunol. 2023 Jan 5;13:1078414. doi: 10.3389/fimmu.2022.1078414 (PMC9849898; doi:10.3389/fimmu.2022.1078414)

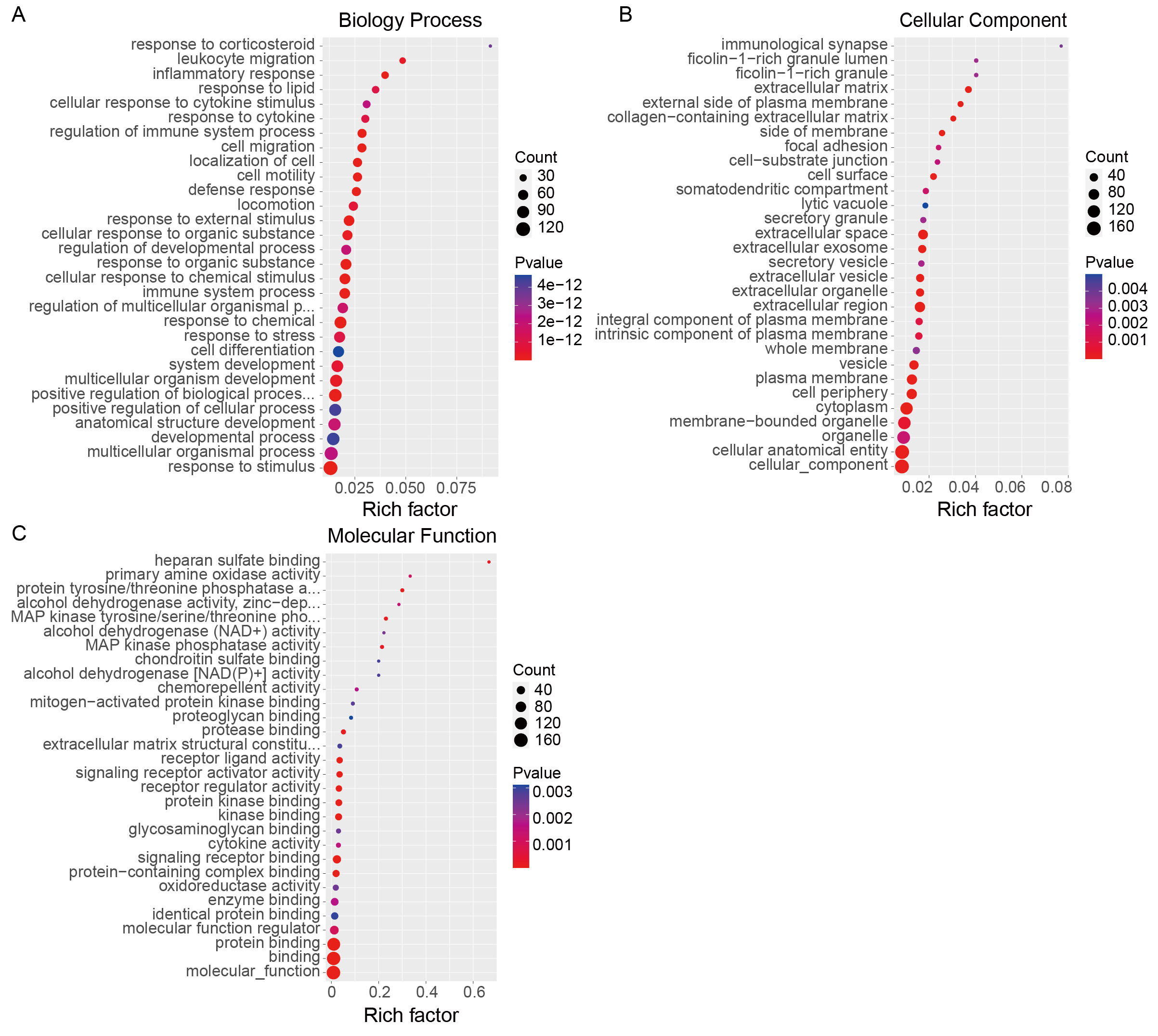

Supplement: Supplementary Figure 1 — GO functional analysis 172 genes associated with OA. (A–C) The GO analysis result showed by biology process, cellular component, and molecular function. [file Image_1.jpeg]

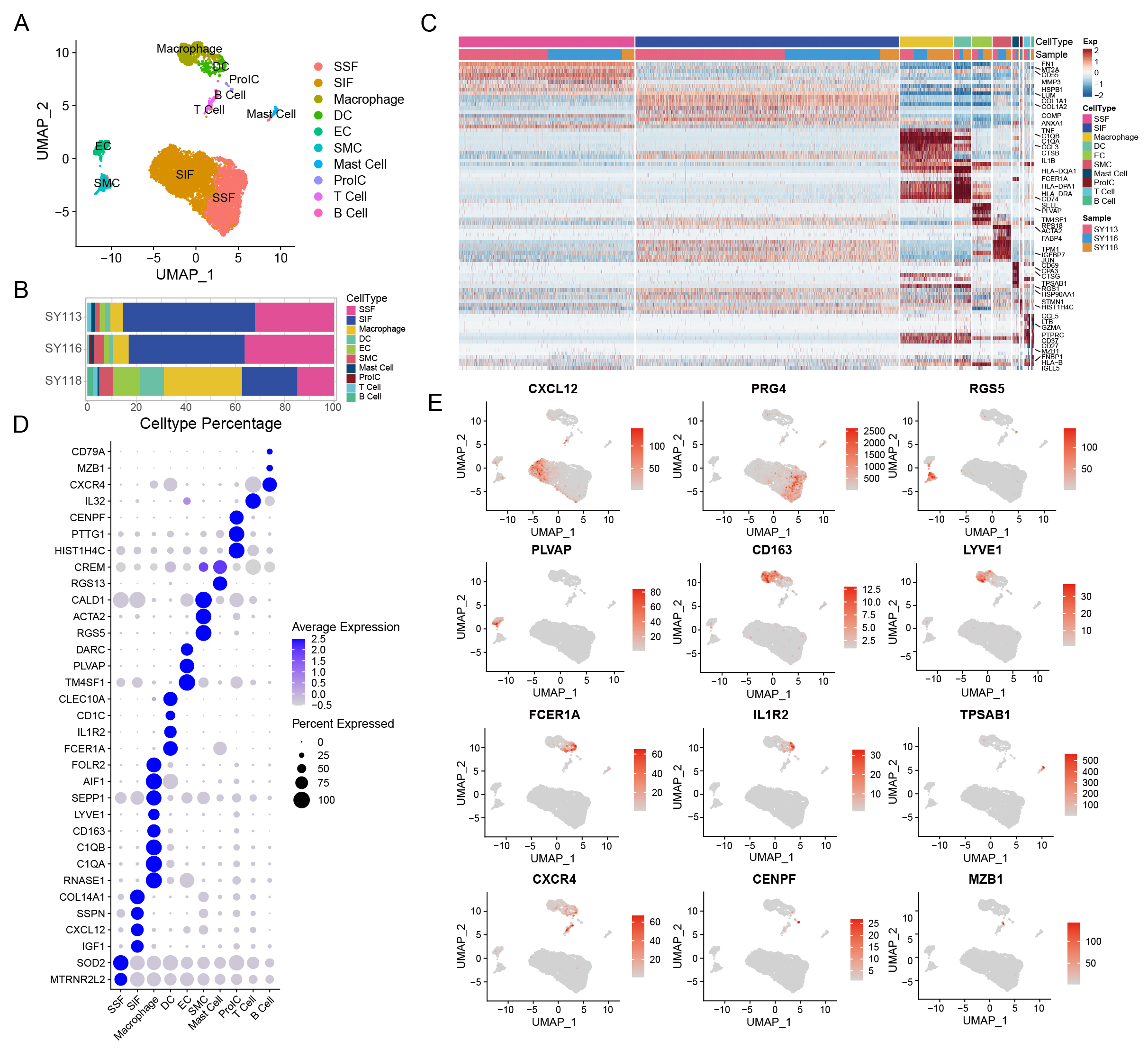

Supplement: Supplementary Figure 2 — Single-cell analysis with three synovial samples of OA. (A) The UMAP showing 10 cell types were identified. (B) The percentage of 10 cell types in each sample. (C) Heatmap of single-cell data based on the UMAP plot. Columns represent individual cells and rows represent genes. (D) The dot plot showing the expression of marker genes in each cell type; the size of the dot reflects the percentage of cells expressing the gene; expression levels are color coded. (E) UMAP plot showing the marker gene expression in all cells. [file Image_2.jpeg]
